# BRCA mutation screening and patterns among high-risk Lebanese subjects

**DOI:** 10.1186/s13053-019-0105-9

**Published:** 2019-01-18

**Authors:** Chantal Farra, Christelle Dagher, Rebecca Badra, Miza Salim Hammoud, Raafat Alameddine, Johnny Awwad, Muhieddine Seoud, Jaber Abbas, Fouad Boulos, Nagi El Saghir, Deborah Mukherji

**Affiliations:** 1 Medical Genetics Unit and Department of Pathology and Laboratory Medicine, American University of Beirut Medical Center, Beirut, Lebanon; 2 Division of Hematology-Oncology, Department of Internal Medicine, American University of Beirut Medical Center, Beirut, Lebanon; 3 Department of Obstetrics and Gynecology, American University of Beirut Medical Center, Beirut, Lebanon; 4 Department of Surgery, American University of Beirut Medical Center, Beirut, Lebanon

**Keywords:** BRCA1, BRCA2, Manchester score, Familial, Lebanon

## Abstract

**Background:**

Previous studies have suggested that the prevalence of *BRCA1* and *2* mutations in the Lebanese population is low despite the observation that the median age of breast cancer diagnosis is significantly lower than European and North American populations. We aimed at reviewing the rates and patterns of *BRCA1/2* mutations found in individuals referred to the medical genetics unit at the American University of Beirut. We also evaluated the performance of clinical prediction tools.

**Methods:**

We retrospectively reviewed the cases of all individuals undergoing *BRCA* mutation testing from April 2011 to May 2016. To put our findings in to context, we conducted a literature review of the most recently published data from the region.

**Results:**

Two-hundred eighty one individuals were referred for testing. The prevalence of mutated *BRCA1* or *2* genes were 6 and 1.4% respectively. Three mutations accounted for 54% of the pathogenic mutations found. The *BRCA1* c.131G > T mutation was found among 5/17 (29%) unrelated subjects with *BRCA1* mutation and is unique to the Lebanese and Palestinian populations. For patients tested between 2014 and 2016, all patients positive for mutations fit the NCCN guidelines for *BRCA* mutation screening. The Manchester Score failed to predict pathogenic mutations.

**Conclusion:**

The *BRCA1* c.131G > T mutation can be considered a founder mutation in the Lebanese population detected among 5/17 (29%) of individuals diagnosed with a mutation in *BRCA1* and among 7/269 families in this cohort. On review of recently published data regarding the landscape of *BRCA* mutations in the Middle East and North Africa, each region appears to have a unique spectrum of mutations.

## Background

Germline mutations in *BRCA1* and *BRCA2* genes have important implications for treatment of patients diagnosed with breast or ovarian cancers as well as unaffected carriers of these mutations. Various guidelines have been established to guide physicians as to which patients should be referred for germline genetic testing. The National Comprehensive Cancer Network (NCCN) guidelines which are developed in the United Sates are freely available to practitioners worldwide and include broad guidelines for mutation testing based on clinical criteria without a calculation of expected mutation frequency.

A number of on-line calculators for risk of mutation assessment, based on clinical data are also available and largely used by clinical genetics specialists. The Manchester Score is a simple scoring system using basic clinical data that has been validated in several European populations to estimate an individual’s mutation risk and determine eligibility for genetic screening.

Patients with breast cancer in the Middle East, particularly in Lebanon, tend to present at a younger age with a median of 50 years compared with the median age at diagnosis of 63 years in Europe and North America [1]. In Lebanon, the prevalence of *BRCA* mutation were reported to be 5.7% among a cohort of patients with breast cancer meeting high-risk criteria [2]. In another study, it was found that 12.5% of referred subjects had deleterious *BRCA* mutation in a cohort of high risk individuals referred for genetic testing [3].

In order to further explore the landscape of *BRCA* mutations in the Lebanese population, we have reviewed all cases referred for *BRCA* mutation testing at the American University of Beirut Medical Center (AUBMC), the largest tertiary referral center in the country. We also aimed at addressing the practice of referral for genetic testing and establishing whether clinician-friendly risk prediction models or guidelines could be helpful in identifying individuals meeting high-risk criteria in our population lacking access to genetic counselors [4], specifically the Manchester Score and NCCN guidelines.

## Methods

After Institutional Review Board (IRB) approval, we retrospectively reviewed the cases of all individuals referred for *BRCA* mutation testing at the Medical Genetics Unit of AUBMC from April 2011 to May 2016. Data regarding family history and the frequency and characteristics of *BRCA* variants were collected from medical charts in order to calculate the Manchester score for this cohort (Table 1) [5]. Receiver Operating Characteristics (ROC) analysis was used to determine the predictive performance of the scoring system to identify those at risk of harboring a pathogenic mutation. Criteria for genetic risk evaluation according to NCCN guidelines were taken from NCCN guidelines version 1.2017 (www.nccn.org).

**Table 1:** Manchester Score

Cancer	Gender	Age at diagnosis	Score
Breast Cancer	Female	< 30	11
Breast Cancer	Female	30–39	8
Breast Cancer	Female	40–49	6
Breast Cancer	Female	50–59	4
Breast Cancer	Female	> 59	2
Breast Cancer	Male	< 60	13
Breast Cancer	Male	> 59	10
Ovarian Cancer	Female	< 60	13
Ovarian Cancer	Female	> 59	10
Pancreatic Cancer	Both		1
Prostate Cancer	Male	< 60	2
Prostate Cancer	Male	> 59	1

To put our findings in the context of the landscape of *BRCA* mutations in the wider Middle East, we conducted a literature review of the most recently published data from the region.

### *BRCA1* and *BRCA2* analysis

Blood samples were collected in ethylenediaminetetraacetic acid (EDTA) tubes, DNA extraction was performed using QiaAmp DNA Mini kit (Qiagen). Amplification of the target genes (*BRCA1* and *BRCA2*) was performed by polymerase chain reaction (PCR) with specific primers designed through Primer 3 [6]. Amplified sequences were visualized on agarose gel 2% to check the efficiency of the PCR. The amplicons were then purified, sequenced and loaded on the Genetic Analyzer (Applied Biosystem ABI 3500). Obtained sequences were analyzed using Seqscape® v2.7 software and compared to the corresponding reference sequences (*BRCA1*: ncbi RefSeq NM_007294; *BRCA2*: ncbi RefSeq NM_000059.3). The significance of each variant found was determined referring to Clinvar database [7].

## Results

### Patients and disease characteristics

We reviewed the results of 281 individuals from 269 families of whom 12 subjects were known to carry a specific mutation diagnosed in an outside laboratory. 97.5% were females and 2.5% were males. The mean age of the cohort was 47.9 years (range 15–86), 208 (74.02%) patients were diagnosed to have breast (194) or ovarian (12) cancer with 2 patients presenting with both simultaneously. The mean age of patients with a diagnosis of breast cancer was 47.7 years (Table 2). Of the families referred because of a known mutation six out of twenty five (24%) of subjects were found to be positive for the specific mutation (Tables 3 and 4).

**Table 2:** Characteristics of patients

Variable	Number/Percentage
Number of patients	281
Number of Families	269
Age (mean, years)	47.86 (15–86)
Positive first-degree family history for Breast and/or Ovarian cancer	165
Positive personal history for Breast and/or Ovarian	208
Breast cancer only	194
Ovarian cancer only	12
Both ovarian and breast cancer	2
Mean age of BC diagnosis (years)	47.71
Families carriers of Deleterious Mutation	10

**Table 3:** *BRCA1* gene mutation and VUS identified in our cohort

Nucleotide Change	AA Change	Nomenclature Protein	Number of families with history of mutation
Deleterious			
c.131G > T	p.Cys44Phe	C44F	7
c.3436_3439delTGTT	p.Cys1146Leufs	3555del4	5
c.2158G > T	p.Glu720Ter	E720X	3
c.3679C > T	p.Gln1227Ter	Q1227X	1
c.679G > T	p.Glu227Ter	E227X	2
c.3381 T > G	p.Tyr1127Ter	Y1127X	3
c.4096 + 1G > A		IVS11 + 1G > A	1
c.1039_1040delCT	p.Leu347Valfs	1158_1159delCT	1
VUS			
c.536A > G	p.Tyr179Cys	Y179C	
c.1456 T > C	p.Phe486Leu	F486 L	4
c.1648A > C	p.Asn550His	N550H	
c.804C > G	p.Asn268Lys	N268K	2
c.3526G > A	p.Val1176Ile	V1176I	1
c.107C > A	p.Ser36Tyr	S36Y	1
c.4132G > A	p.Val1378Ile	V1378I	2
c.3956G > T	p.Gly1319Val	G1319 V	1
c.2617 T > C	p.Ser873Pro	S873P	1
c.488G > C	p.Arg163Thr	R163T	1
c.346G > A	p.Glu116Lys	E116K	1
c.1717_1717delT	p.Ser573HisFsX	1863delT	1

**Table 4:** *BRCA2* gene mutation and VUS identified in our cohort

Nucleotide Change	AA Change	Nomenclature Protein	Number of families with history of mutation
Deleterious			
c.9257-1G > A		IVS24-1G > A	3
c.3189_3192delGTCA	p.Ser1064Leufs	3417del4	1
c.426-12_426-8del5		IVS4-12del5	1
VUS			
c.8775G > C	p.Gln2925His	Q2925H	1
c.1627C > A	p.His543Asn	H543N	1
c.8687G > A	p.Arg2896His	R2896H	
c.8548G > A	p.Glu2850Lys	E2850K	2
c.632-5 T > C		IVS7-5 T > C	1
c.9117 + 3A > G		IVS23 + 3A > G	1
c.3131G > T	p.Cys1044Phe	C1044F	1
c.7976 + 49A > G		IVS17 + 49A > G	1

### Mutations

In this cohort of 281 patients referred for *BRCA* testing at AUBMC, we found 17 patients with *BRCA1* mutations and 4 patients with *BRCA2* mutations, as seen in Table 3 and Table 4. Three common mutations (two *BRCA1* and one *BRCA2*) accounted for 54% of the pathogenic mutations found in this cohort.

The most common *BRCA1* mutation, c.131G > T was found in 5 patients and is carried by 7 Lebanese families (2 patients with family history positive of the c.131G > T mutation were found to be negative for the mutation). *BRCA1* 3555del4 mutation was also common, found in 4 patients and carried by 5 families. *BRCA1* E720X (c.2158G > T), ranked third in frequency and was present in 2 patient and found in 3 families (Table 3). In the *BRCA2* gene, the mutation IVS24-1G > A, was found in 2 patients and carried by 3 families (Table 4.) A number of Variant of Undetermined Significance (VUS) were identified in *BRCA1* and *2* (Tables 3 and 4).

### NCCN guidelines and Manchester scores

**Fig. 1:**
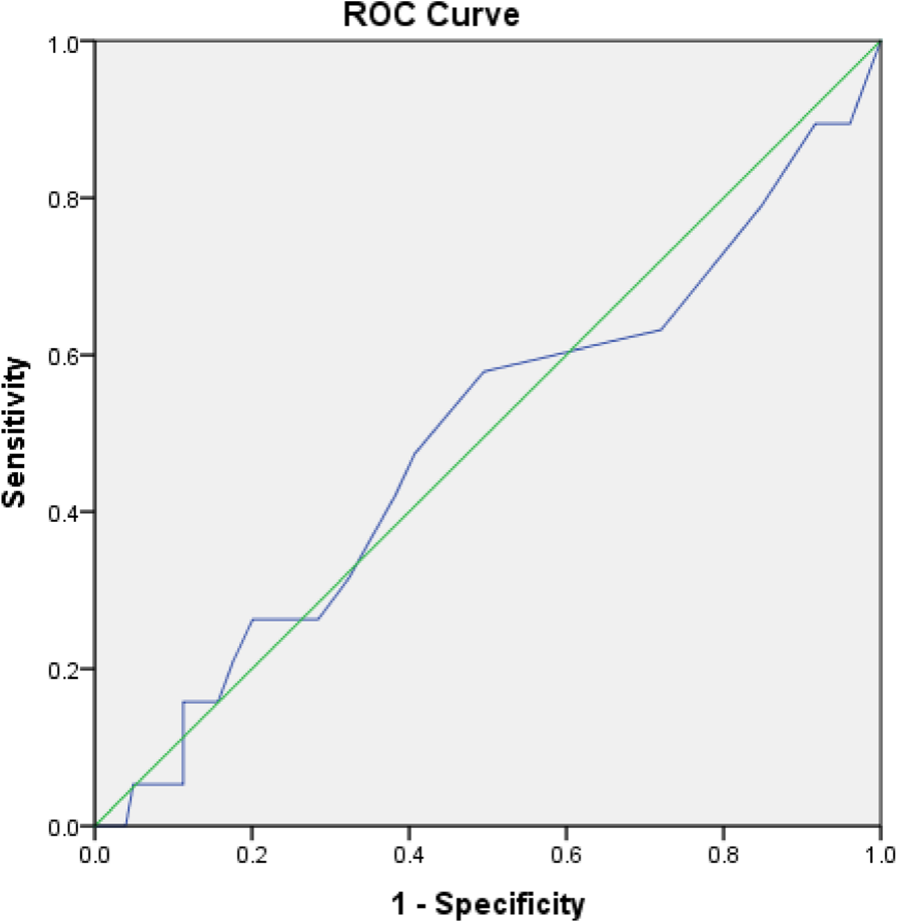
Performance of Manchester score in predicting likelihood of *BRCA* mutation:  The area under the ROC curve of 0.494 (95% CI 0.349–0.639)

For the cohort of patients tested between 2014 and 2016 detailed family history was available. All patients positive for *BRCA1* or *2* mutations fit the NCCN guidelines for *BRCA* mutation screening. The Manchester Score for all tested individuals was calculated, it did not discriminate between positive and negative *BRCA* mutation test results in this cohort with the area under the ROC curve of 0.494 (95% CI 0.349–0.639). Mean Manchester Score in patients negative for a *BRCA* mutation was 12.15 and that of patients positive for *BRCA 1/2* mutations was 11.63 (Fig. 1).

### Literature review of *BRCA* mutations in the MENA region

In order to put our data in to context, we reviewed the spectrum of mutations reported in the Middle East and North Africa (MENA) region, updating a previous review published in 2015 [8].

Studies conducted in the MENA region in the past 3 years have reported a number of new mutations especially in Moroccan, Tunisian, Jordanian and Lebanese families. The most common mutations found in the MENA region are included in the Tables 5 and 6 below.

**Table 5:** Studies examining *BRCA1* & *2* mutations in Middle East and North Africa region from 2015 to 2018

Region	Case selection	Gene region covered	Detection method	Reference
MENA	173 breast cancer and ovarian cancer	All	Parallel sequencing Sanger sequencing	[17]
Lebanon	250 females	All	Sequencing MLPA	[2]
Lebanon	45 families	All	Sequencing Sanger validation	[15]
Tunisia	7 families	All	Sequencing	[18]
Tunisia	92 families		Review	[11]
Jordan	100 BC females	All	*BRCA* sequencing	[10]
Saudi Arabia	818 BC patients	All	Capture or Sanger sequencing	[19]
Morocco	6 families - 15 patients	All	Next generation sequencing	[13]
Morocco	40 patients	All	Sequencing	[12]
Morocco	122 patients	c.1310_1313delAAGA	Sanger sequencing Next generation sequencing	[20]
Palestine	875 patients	BROCA panel	Parallel Sequencing	[14]
Saudi Arabia	310 patients	*BRCA1* and *BRCA2*	Next generation sequencing Sanger sequencing	[9]

**Table 6:** Common genetic variant in the MENA region

*BRCA1* or *2*	Genetic Variant	Number of patients with the mutation	Reference
	Jordan		[10]
*BRCA2*	c.2254_2257delGACT	4	
	c.6685G > T	2	
	Dup exon 5–11	4	
	c.8774A > G	2	
	K3416E	2	
	Tunisia		[11, 13]
*BRCA1*	c.211dupA	8	
	c.5266dupC	5	
	c.1309del4	4	
	c.4041delAG	2	
	c.798_799delTT	2 families	
*BRCA2*	c.1542_1547delAAGA	2	
	c.7887_7888insA	2	
	c.1309del4	5	
	Morocco		[12, 13]
*BRCA1*	c.798_799delTT	2	
	c.5062_5064delGTT	2	
*BRCA2*	c.7710delA	2	
	c.7235insG	7	
	Algeria		[13]
*BRCA1*	c.798_799delTT	2 families	
	Palestine		[14]
*BRCA2*	c.2482delGACT	6	
	Lebanon		[2]& [3, 15]
*BRCA1*	c.131G > T	6	
*BRCA2*	c.9257_1G > A	3	
	Saudi Arabia		[9]
*BRCA1*	c.4136_4137delCT	5	
	c.4524G > A	5	

**Fig. 2:**
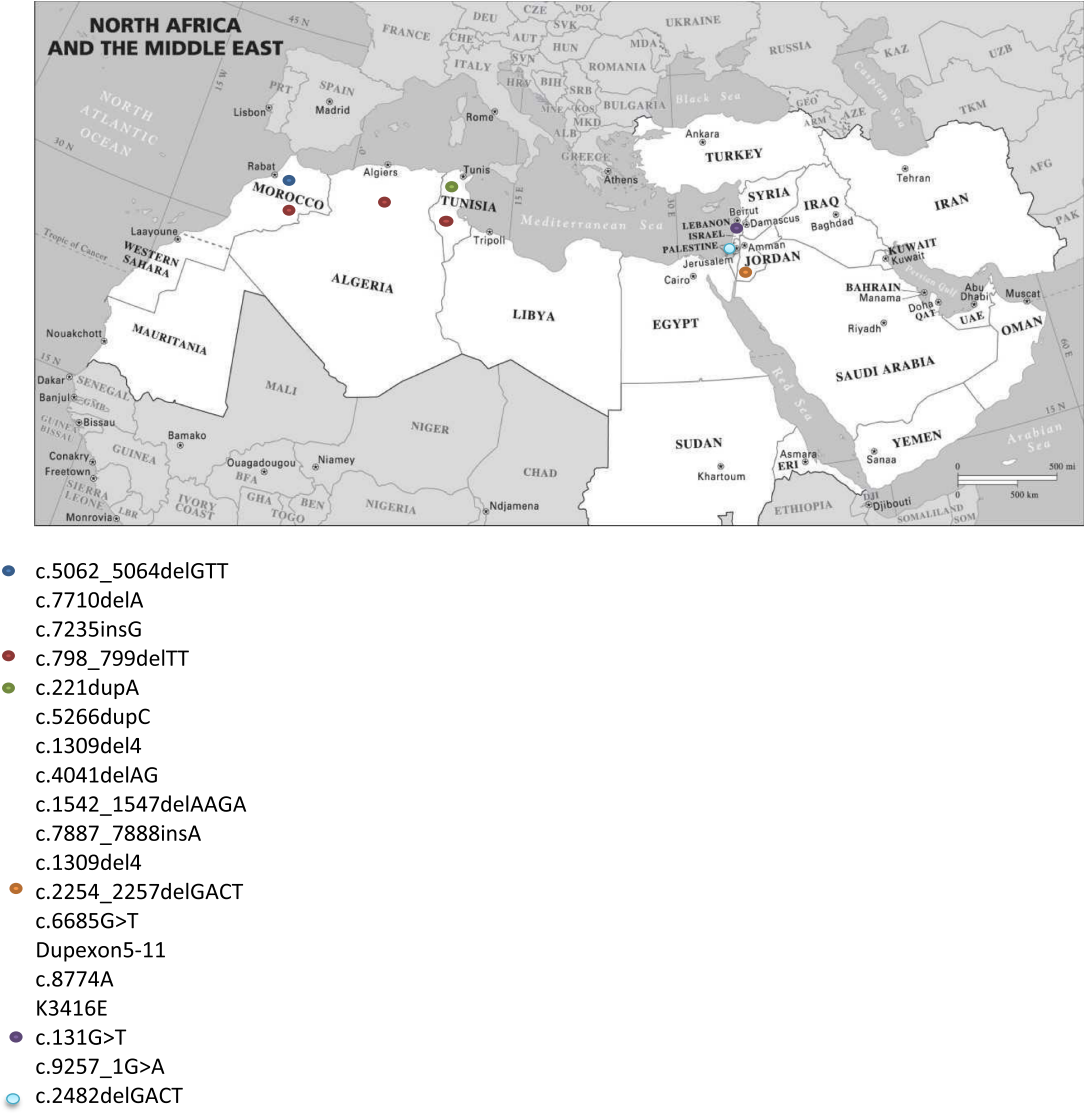
Map of the MENA region with the different *BRCA* mutations identified

In Saudi Arabia, the *BRCA1* c.4136_4137delCT and c.4524G > A were identified as the two most common mutations each found in 5 patients out of 310 enrolled in the study, accounting for 30.4% of the total *BRCA* mutations found in the cohort tested [9]. In Jordan, the *BRCA2* c.2254_2257delGACT and Dup exon 5–11 were the 2 most common mutations each found in 4 patients out of 100 females enrolled in the study, accounting for 40% of patients presenting with the *BRCA* mutation [10, 11]. In Tunisia, the c.211dupA was the most common mutation found in 8 patients out of 92 from unrelated families, 29.6% of all the *BRCA* mutations found in this study [11]. In Morocco, several mutations were found, the most common being the c.7235insG in 2 different patients out of 40 study participants, 18.2% of the *BRCA* mutation carriers [12] . The *BRCA1* c.798_799delTT was a common mutation in North Africa [13], however this has not been identified in studies conducted in the Middle East, namely Lebanon, Jordan or Palestine. This mutation is the only one found in several countries in the MENA region, all the other new mutations were exclusive to one country with the exception of the c.131G > T found in Lebanon and in one Palestinian patient [14] (Fig. 2).

## Discussion

In a population of high-risk individuals referred for genetic testing at our institution, the prevalence of *BRCA1/2* mutation was 7.8%. A c.131G > T mutation was detected among 5/17 (28%) of individuals found to have a mutation in *BRCA1* and among 7/28 (25%) families. The c.131G > T mutation has only recently been classified as pathogenic [7] and can be considered as a founder mutation in the Lebanese population. It is present in a relatively large number of families in our study and has previously identified in other studies conducted in Lebanon [2, 3, 15], and in a study conducted in Palestinians [14]. All individuals referred for genetic testing fit the broad NCCN guidelines however in a resource-limited health-care system where genetic testing is not usually covered by third-party payers, as is the situation in Lebanon and many other countries, identifying the individuals at highest risk for priority testing is desirable. The *BRCA1* c.131G > T and c.3436_3439delTGTT along with the *BRCA2* mutations account for 54% of the total *BRCA* mutations found in our cohort. In a resource limited environment, if the frequency of these mutations is validated in larger studies we could consider a cost-effective targeted screening test for these 3 common mutations as had been proposed in an Italian population harboring a founder mutation [16].

The Lebanese population has limited access to clinical genetics specialists and information regarding genetic testing is usually delivered by other health-care professionals such as oncologists [4]. The Manchester Score is a simple and accessible scoring system that has been used in Europe to identify individuals at high risk of harboring a *BRCA* mutation but has not been validated in the Middle Eastern population were the median age of diagnosis of breast cancer is significantly lower. In this cohort the Manchester score failed to discriminate between positive and negative tests. One of the limitations of this study is the size of the cohort and low absolute number of individuals tested and mutations identified. Another limitation is the retrospective nature of the data collection, although patients were prospectively assessed by a clinical geneticist at the time of testing. The relatively low rate of mutations in the *BRCA1* and *2* genes in this high-risk population with breast cancer diagnosed in the relatively young, suggests that mutations in other genes may be prevalent.

To give a broader perspective on our results, we reviewed the reported mutations in the MENA region (Tables 5 and 6, Fig. 2). The founder mutation identified in our cohort seems to be unique to the Lebanese/Palestinian populations. An interesting finding is the mutual exclusivity of reported founder mutations in each of the countries in the region, with the exception of the *BRCA1* c.798_799delTT identified in several North African populations.

## Conclusion

In a cohort of high-risk Lebanese individuals referred for genetic testing, pathogenic *BRCA1/2* mutations were detected in 7.8%. While all subjects fit current NCCN guidelines for testing, the Manchester score failed to discriminate between those with positive and negative test results in this study. The *BRCA1* mutation c.131G > T was detected among 5/17 (29%) of individuals found to have a pathogenic *BRCA1* mutation in the cohort and can be considered a founder mutation in the Lebanese population. On review of recently published data regarding the landscape of *BRCA* mutations in the MENA, each region appears to have a unique spectrum of mutations. Further investigation of other genes involved in young-onset and familial breast cancer in Lebanon are ongoing.

## Abbreviations

AUBMC: American University of Beirut Medical Center
EDTA: Ethylenediaminetetraacetic acid
IRB: Institutional review board
MENA: Middle East and North Africa
NCCN: National comprehensive cancer network
PCR: Polymerase chain reaction
ROC: Receiver operating characteristics
VUS: Variant of undetermined significance
